# A four-component model of the action potential in mouse detrusor smooth muscle cell

**DOI:** 10.1371/journal.pone.0190016

**Published:** 2018-01-19

**Authors:** Mithun Padmakumar, Keith L. Brain, John S. Young, Rohit Manchanda

**Affiliations:** 1 Department of Biosciences and Bioengineering, Indian Institute of Technology Bombay, Mumbai, India; 2 Institute of Clinical Sciences, College of Medical and Dental Sciences, University of Birmingham, Birmingham, United Kingdom; 3 School of Pharmacy and Biomedical Sciences, University of Portsmouth, Portsmouth, United Kingdom; Cinvestav-IPN, MEXICO

## Abstract

**Background and hypothesis:**

Detrusor smooth muscle cells (DSMCs) of the urinary bladder are electrically connected to one another via gap junctions and form a three dimensional syncytium. DSMCs exhibit spontaneous electrical activity, including passive depolarizations and action potentials. The shapes of spontaneous action potentials (sAPs) observed from a single DSM cell can vary widely. The biophysical origins of this variability, and the precise components which contribute to the complex shapes observed are not known. To address these questions, the basic components which constitute the sAPs were investigated. We hypothesized that linear combinations of scaled versions of these basic components can produce sAP shapes observed in the syncytium.

**Methods and results:**

The basic components were identified as spontaneous evoked junction potentials (sEJP), native AP (nAP), slow after hyperpolarization (sAHP) and very slow after hyperpolarization (vsAHP). The experimental recordings were grouped into two sets: a training data set and a testing data set. A training set was used to estimate the components, and a test set to evaluate the efficiency of the estimated components. We found that a linear combination of the identified components when appropriately amplified and time shifted replicated various AP shapes to a high degree of similarity, as quantified by the root mean square error (RMSE) measure.

**Conclusions:**

We conclude that the four basic components—sEJP, nAP, sAHP, and vsAHP—identified and isolated in this work are necessary and sufficient to replicate all varieties of the sAPs recorded experimentally in DSMCs. This model has the potential to generate testable hypotheses that can help identify the physiological processes underlying various features of the sAPs. Further, this model also provides a means to classify the sAPs into various shape classes.

## 1 Introduction

The detrusor smooth muscle (DSM) located in the mammalian urinary bladder wall controls the bladder tone, aiding the storage and micturition of urine. DSM cells (DSMCs) are electrically connected to one another via gap junctions and form a three dimensional electrical syncytium [1–6]. It is innervated by the sympathetic and parasympathetic nerves [1, 2]. Neurotransmitters that regulate DSM contractions are released from special structures known as varicosities, which are small swellings along the axons that supply the tissue [3, 7]. Multiple varicosities occur on the same axon. Single axon supplies multiple DSMCs and several axons can excite the same DSMC. Due to this many-to-many mapping of the DSMCs and the neurotransmitter release sites, and also due to the electrically interconnected DSM syncytium, the DSMCs exhibit complex electrical activity. The electrical activity observed in DSMC is difficult to analyze, e.g., with respect to (i) sources of activity and (ii) constituent components of signals observed in the cells.

It can be observed from electrophysiological experiments on isolated DSM strip preparations that most DSMCs exhibit spontaneous electrical activity [8–12]. Such activity consist principally of passive signals called spontaneous transient depolarizations and active signals called spontaneous action potentials (sAPs). It is established that in the mouse, spontaneous transient depolarizations are produced by the spontaneous release of neurotransmitter vesicles from the varicosities and hence could be termed as spontaneous excitatory junction potentials (sEJPs) as in the case with the vas deferens. These vesicle release events are random and may not be preceded by an AP in the parent nerve terminal. The sEJPs thus evoked in the target DSMC exhibit finely graded amplitudes [3, 10, 11]. If an sEJP has amplitude greater than the threshold for spike firing, it results in a sAP.

**Fig 1:**
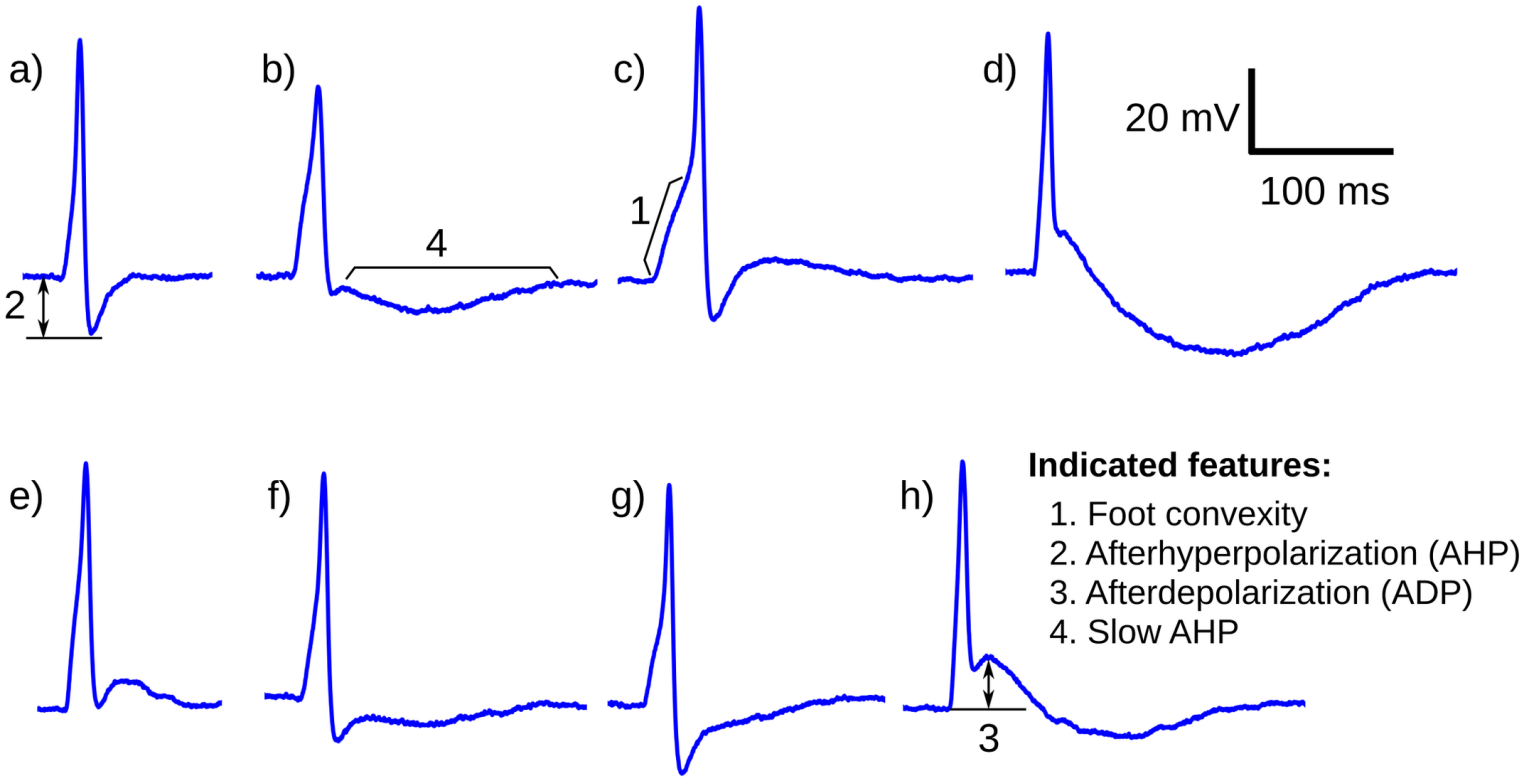
One example each from different sAP shapes observed from a typical detrusor smooth muscle cell during an electrophysiological recording session. The four key features—1) foot convexity, 2) after hyperpolarization (AHP) 3) afterdepolarization (ADP), and 4) slow AHP—which distinguish the shape differences are also indicated.

The mouse DSMC exhibit spontaneous APs of both neurogenic and myogenic origin [10, 11, 13–15]. The sAP with a neurogenic origin was termed type A AP and the ones with myogenic origin was termed type B AP. The type B APs are also called pacemaking APs [10, 11] and has a signature shape. However, it is observed that the shapes of the type A sAPs recorded from a single DSMC can vary widely [11, 16–19]. This is in sharp contrast to the property of excitable cells belonging to other tissues such as cardiac and skeletal muscles, and almost all kinds of neurons—where a signature AP shape is produced by any single excitable cell throughout the recording. So far these shape variations in Type A sAPs were not studied in detail. Our investigation addresses this gap. An example of the set of sAP shapes observed from a single DSMC is shown in Fig 1. These differences in AP shapes are distinguished by the variations in its key features such as the degree of convexity at the initiation of an AP, magnitude and kinetics of the after hyperpolarization (AHP), the after depolarization (ADP), and slow after hyperpolarization (sAHP), which are marked in the figure. The biophysical reasons that trigger the variations in these features are currently unknown. As it seems unlikely that the membrane properties of the cell changes spontaneously during a session of recording, the possible reason for this shape variety could be associated to the properties of the complex syncytial arrangement of the DSMCs and distributed innervations.

**Fig 2:**
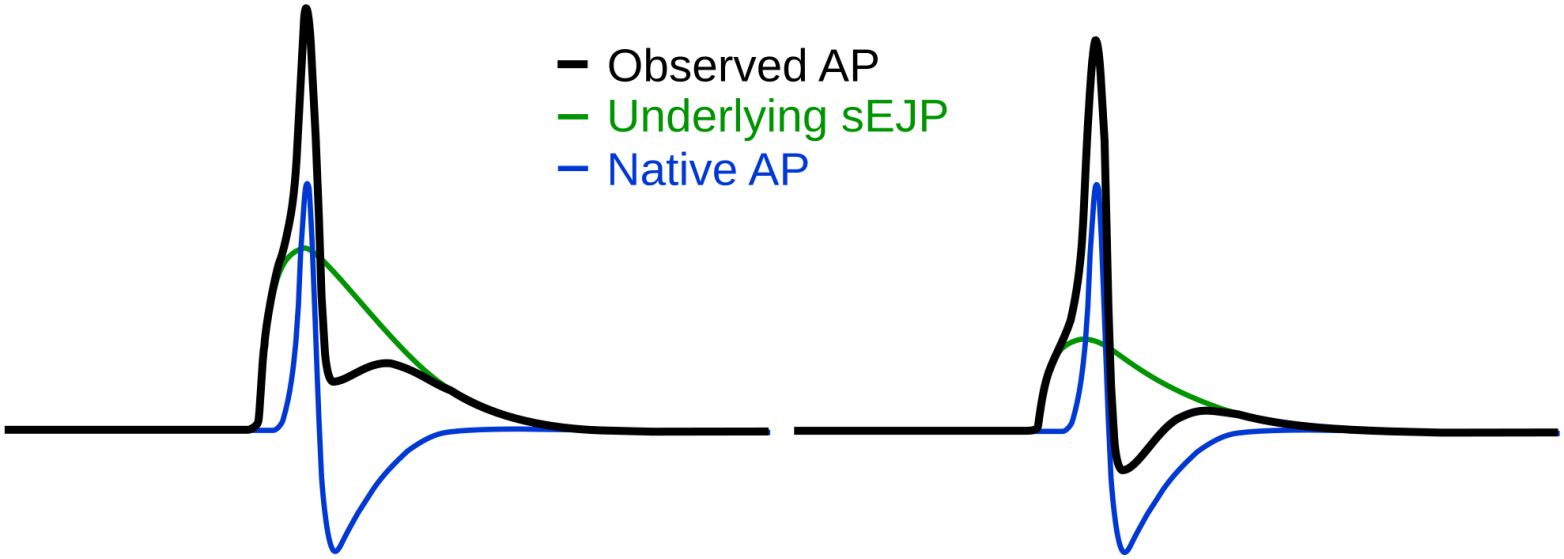
Schematic figure depicting the hypothesis that the variations in AP shape is caused by an underlying sEJP [16]. Note that when the amplitude of the underlying sEJP is larger (left) the resultant AP has a taller foot, larger afterdepolarization and no afterhyperpolarization.

One hypothesis explaining the shape varieties in sAPs observed in the DSMCs is the sEJP superposition hypothesis (Fig 2) [16]. There, it is proposed that there exists a variable superposition between the sEJP initiated by the neurotransmitter action, which depolarized the cell membrane above the threshold, and the AP generated by the voltage-gated ion channels of the cell. If such a superposition exists, the features of the sAPs would be correlated, and not independent. A pilot study conducted on similar lines [16] has shown that there is a significant correlation between the foot and the tail features of the sAPs.

The sEJP superposition hypothesis could explain the shape variations in a subset of observed sAPs, but not all of them. This indicates that there might exists more components other than the sEJP which superimpose with the native AP that is produced in the DSMC. In this work, we try to identify all the major sub components which are superposed to obtain the sAP shape observed from the DSMC. To achieve this, we have adopted a novel approach which involves the comparison of the various features of the signals observed from the intracellular electrical recordings from the DSMCs. If two signals with different shapes were observed to have a set of matching features, there is a possibility that one of those signals contain an extra component which produced the shape difference between them, and efforts are made to extract that extra component by subtracting one signal from the other. Such an approach has not been used before for exploring a smooth muscle syncytium.

The shapes of the sAPs carry important information related to the syncytial properties and cellular biophysical properties of the detrusor. For example, the nature of the sAP foot provides information as regards to (i) the source of the sAPs in relation to the recording location, (ii) the degree of propagation of the sAPs (and deriving from this, an estimate of the size of the smooth muscle bundle in which the sAP is propagating), and (iii) the origin of the sAPs, i.e., whether they are neurogenic or myogenic [10, 11]. The properties of sAP tails, such as the presence or absence of long afterhyperpolarization influences the refractory period of the sAP thereby controlling the maximum AP frequency. Thus, analysis of sAP shapes can provide a variety of insights into the biophysics of the tissue.

## 2 Methods

### 2.1 Ethics statement

The Institutional Animal Care and Use Committee (IACUC) covering the Department of Pharmacology, University of Oxford, approved and had oversight of all animal experiments. All recordings were obtained during or before 2013, and were approved under Animals (Scientific Procedures) Act 1986, but are also consistent with both UK Animals (Scientific Procedures) Act (2013) and European Communities Council Directive 2010/63/EU.

### 2.2 Electrophysiological recordings

The electrophysiological recordings used in this study follows the protocol described in our earlier works [10, 11]. Briefly, C57BL/6 strain mice were sacrificed by head concussion followed by cervical dislocation. Strips of bladder wall were maintained at 35°C in a bicarbonate-buffered physiological saline, with muscle cells recorded from using sharp (100 − 300 MΩ) microelectrodes, with a high-impedance headstage and an Axoclamp-2B used in Bridge mode. This electrophysiology configuration allows recording of the normal changes in the cells’ membrane potential over time, aiming to minimize any perturbation induced by the process of recording.

### 2.3 Identification of the components

**Fig 3:**
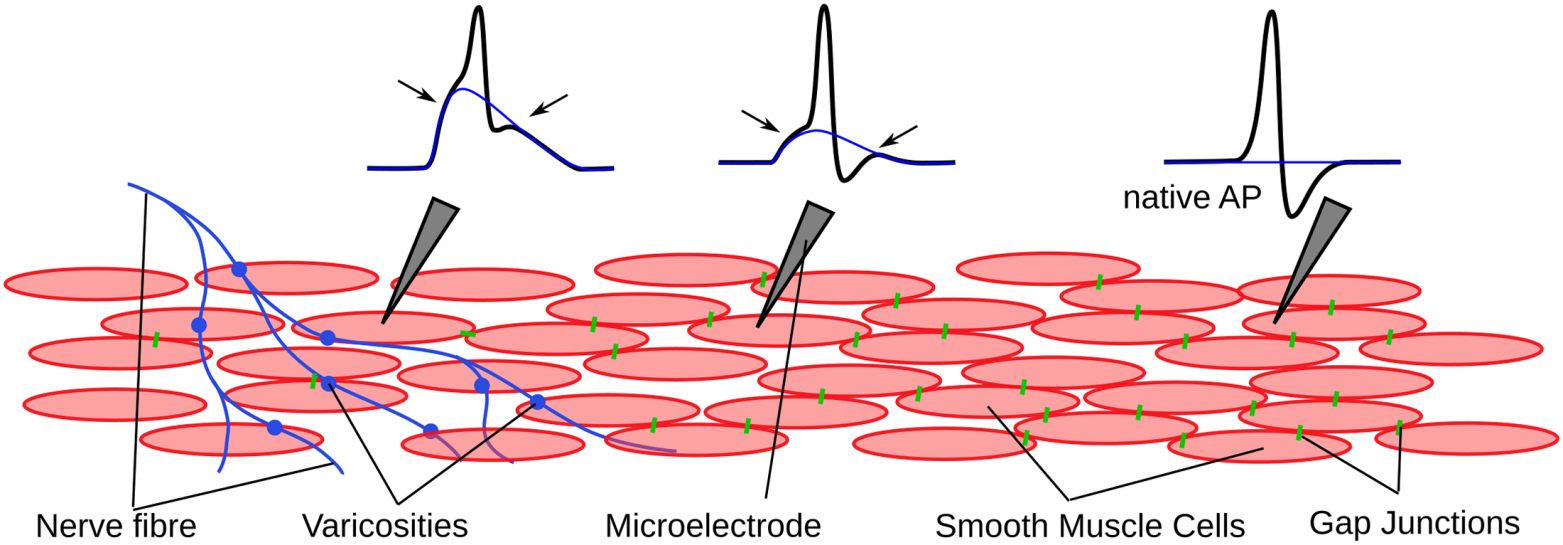
A schematic diagram showing the attenuation of the AP foot convexity and after depolarization amplitude based on distance from the varicosity. The smooth muscle cells, connected via the gap junctions allow the unattenuated active transmission of APs (shown in black trace). The underlying sEJP (shown in blue trace) is passive signal and is attenuated with distance. The reduced amplitude of the underlying sEJP over distance is visible in the foot and the ADP amplitude (indicated by the arrows) of the observed sAP. At a sufficiently large distance, the underlying sEJP disappears and the observed AP attains the shape of the native AP.

The passive (i.e. non-regenerative) depolarization of membrane potential caused by the excitatory neurotransmitter action is seen as the convex foot of the APs recorded at the vicinity of the varicosity [20]. This convexity becomes less evident as the distance between the varicosity and the site of recording increases, because of the attenuation of the passive potential. Eventually when the distance becomes more than 4-5 times the space constant of the cell membrane, the convex foot of the AP is lost and it turns concave (Fig 3). This kind of AP, generated by local circuit currents, is termed the native AP (Fig 3) which is not contaminated by an sEJP signal [16]. The APs present in the spontaneous electrical activity of DSMC have predominantly convex feet. It could be inferred that a convex-foot AP observed from the DSMC contains two basic components—1) the neurotransmitter-evoked passive component and 2) the uncontaminated native AP or nAP.

The isolated sEJPs seen in the recordings could be identified as the former component. The individual sub threshold sEJPs seen in the intracellular recordings are the neurotransmitter release events which failed to elevate the membrane potential to the threshold value. The events which generate the sEJPs with amplitude greater than the threshold would cause an AP, and the generated AP would be superimposed on the sEJP—which is expressed as the convex foot and ADP of the AP.

**Fig 4:**
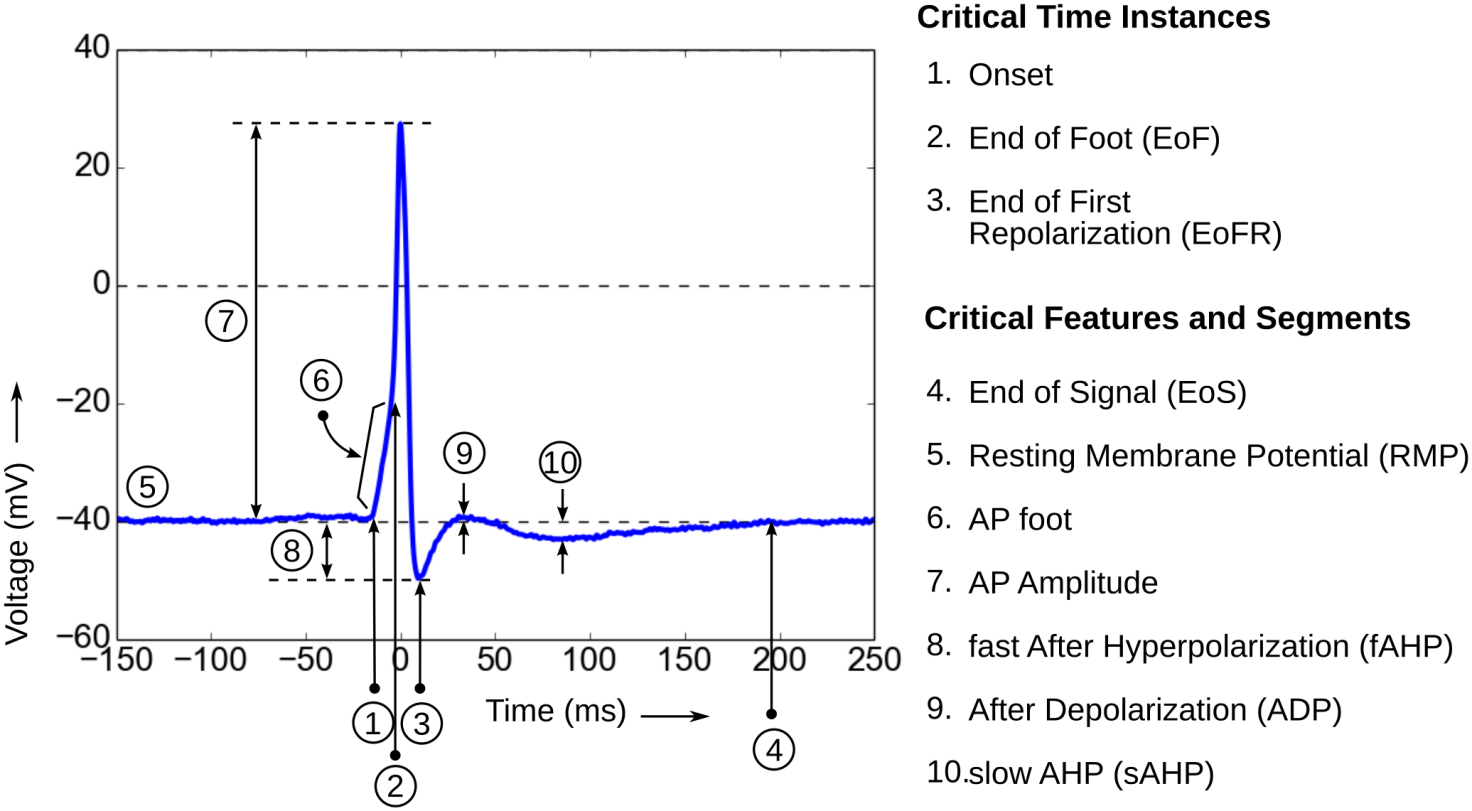
Features and terminology used in the work indicated on a sample AP.

The membrane potential of the hypothetical native AP (Schematically shown in Figs 2 and 3) falls to a hyperpolarized value after its peak and then slowly go back to the resting state. It is expected that the follow-through of the native AP to the resting state after the end of first repolarization (EoFR, see Fig 4) is monotonic. The presence of sEJP underlying the nAP causes the shape of the nAP to have variations and exhibit convex feet and ADP.

**Fig 5:**
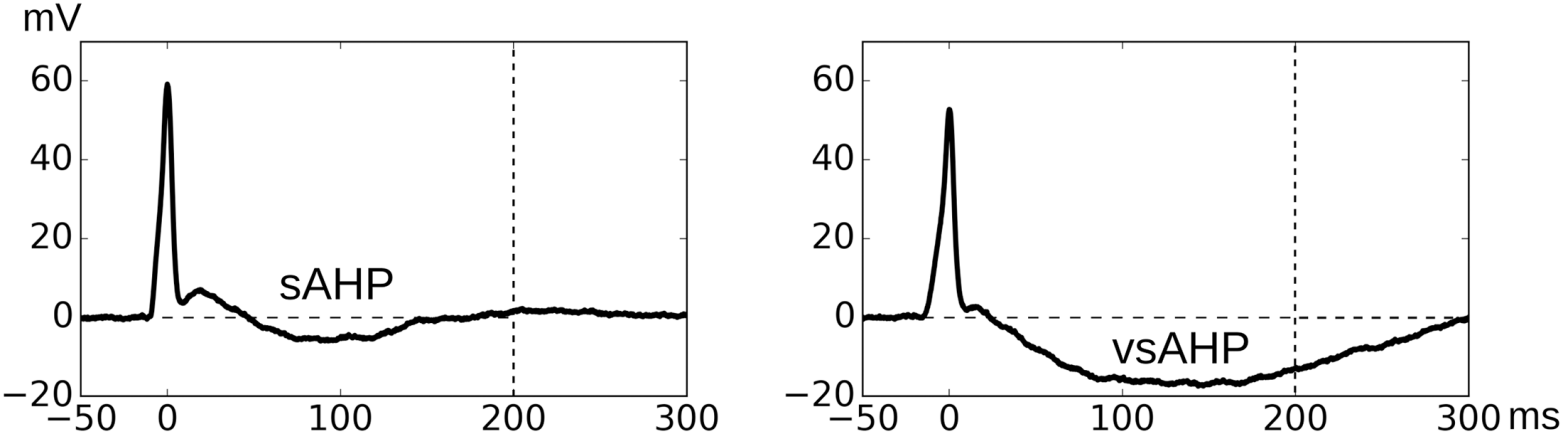
One example each for AP signals displaying (left) slow afterhyperpolarization (sAHP) and (right) very slow afterhyperpolarization (vsAHP). Note the difference in amplitudes and time spans of the hyperpolarization. The presence of slow AHP in an AP cannot be explained by the underlying-sEJP hypothesis, indicating the presence of more components.

However, superposition of an sEJP shape with the nAPs cannot not produce all of the diverse shapes of detrusor sAPs, especially those with the slow AHP (sAHP—indicated by Feature 9 in Fig 4). An AHP is said to be an sAHP if it has a time course ≥ 100 ms. APs with sAHP are very commonly observed in the DSMC cells (40%). The typical duration of sAHPs observed in sAPs is about 200 ms. Though it is currently established that the blockade of small conductance Ca^2+^-activated K^+^ channels (SK channels) abolish the sAHP feature [15, 21], the trigger which activates or deactivates SK channels during the spontaneous activity of DSMC is currently unknown. However, there are other type of sAHPs observed in sAPs with a duration of about 300 ms. These are two distinct populations with no intermediate population of sAHPs with a graded duration between them. This indicated that two distinct mechanisms might be involved in generating these two types of sAHPs. To differentiate these two, the latter population of sAHPs with higher amplitude and longer duration is termed as very slow AHP (vsAHP). The existence of vsAHP in DSMCs have not been addressed in the literature so far. Two sample APs, one with sAHP and the other with vsAHP are shown in Fig 5.

On the basis of the above mentioned observations, it was concluded that the four components that constitute an sAP exhibited by DSMC are: (i) sEJP, (ii) native AP (or nAP), (iii) sAHP, and (iv) vsAHP. Also, we propose the hypothesis that any neurogenic AP observed in DSMC could be represented to a first level of approximation as a linear combination of these four components.

### 2.4 Isolation of sEJP and nAP

**Fig 6:**
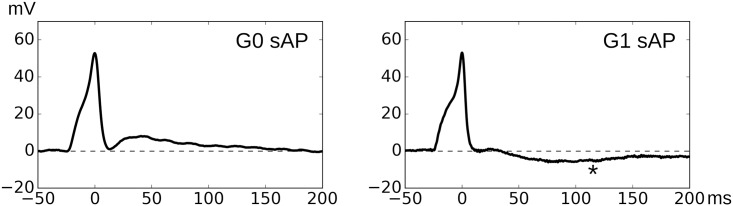
One example each for AP signals of Group 0 (G0, left) and Group 1 (G1, right) categories. Note the absence of the slow AHP component (marked using * in right panel) in G0 AP, aiding our assumption that such APs consist of only two components—sEJPs and native APs.

The neurogenic sAPs are classified into two groups G0 and G1. Group 0 or G0 APs, which do not express any type of sAHPs, and Group 1 or G1 APs, which express sAHP and/or vsAHP. Example of APs from G0 and G1 are shown in Fig 6. For the estimation of nAP, G0 APs are used. It is assumed that the G0 APs have only two components present in them, the sEJP and the nAP. It is possible to derive a prototype of the sEJP component using the sEJP shapes observed from the same cell. We operate from the framework that neurotransmitter action underlying the generation of sEJPs and sAPs is the same, except that during the generation of sEJP alone, the threshold voltage of the DSMC is not reached. Thus it may be possible to represent the sEJP underlying the AP by an amplified version of the closely matching sEJPs observed spontaneously in the same cell.

A two step approach was followed to obtain the underlying sEJP from an AP. For every sEJP present in the cell, 1) The onset of sEJP was aligned with that of AP, and (2) A multiplier which minimizes the distance measure obtained between the foot of the AP and the corresponding section of sEJP was obtained. The process was repeated for every sEJPs recorded in the cell and the sEJP which returns the minimum distance from the AP foot was considered to be the closest approximation of the underlying sEJP for that AP. The sEJP approximation thus obtained was considered as the first component. Since the G0 APs contain only two components, the second component, namely the nAP was obtained by subtracting the sEJP approximation from the corresponding AP. The algorithms used for the onset detection and subsequent evaluation of the sEJP multiplier are given below.

#### 2.4.1 Onset detection

We defined the onset of a signal as the instant when the membrane potential reaches > RMP + *σ*_*n*_ where *σ*_*n*_ is the standard deviation of the biological noise present in the baseline of the signal. As the different signal recordings used in the study had different noise levels, a hard threshold of $\text{RMP}+max(\sigma_{n_{i}})$ mV, where $\sigma_{n_{i}}$ is the SD of noise for the *i*th signal, was used for the estimation of the signal onset.

#### 2.4.2 The distance measure

To obtain the distance measure between an AP foot and an sEJP, the foot of the AP was first extracted using the slope analysis. The slope profile of the convex-foot AP shows a double peak during its rising phase [10, 11]. The instant at which the slope reaches the valley between these peaks is taken as the end of the foot (EoF). The AP foot vector was defined as the segment of the AP between its onset point and the EoF. This vector was represented by the variable *V*_*f*_. A section of the same length was extracted from the rising part of the matching sEJP starting from its onset location. This vector was identified by the variable *V*_*s*_. Now the distance between the AP and the sEJP was measured using the following formula:

$$\begin{array}{c} D=min(\text{Distance}\left(V_{f},m*V_{s}\right),\forall m\in \left(1,1.5\right)) \end{array}$$ (1)

Where *m* is the multiplier for the sEJP vector *V*_*s*_. The value of *m* is limited to an interval (1, 1.5) as it is unlikely that the shape of an sEJP is maintained for a multiplier outside that window. The function Distance(*a*, *b*) is the Euclidean distance measure between the vectors *a* and *b*. A range of multiplier values (*m*_*i*_, ∀*i* = 1, 2, 3, …, 1000) were used to estimate the distance between the vectors *V*_*f*_ and *V*_*s*_ and the multiplier which gave the minimum distance was finalized as the best multiplier for the sEJP. However, the partially active behavior of the AP foot could cause the value obtained for *m* go higher than that of the ideal multiplier. This needs to be avoided by giving a greater weight to the initial part of the foot.

In order to do so, the vectors *V*_*f*_ and *V*_*s*_ were divided into two halves—*V*_1*f*_ and *V*_2*f*_, and *V*_1*s*_ and *V*_2*s*_ respectively. The distance measured (*D*) between *V*_*f*_ and *V*_*s*_ was then expressed as a sum of sub components *d*1 and *d*2 where

$$\begin{array}{cc} & d1=\left|\right|V_{1a}-m_{i}*V_{1s}\left|\right| \end{array}$$ (2)

$$d2=\sqrt{\underset{k}{\sum }{\left(\frac{V_{2f}\left(k\right)-m_{i}*V_{2s}\left(k\right)}{V_{2f}\left(k\right)}\right)}^{2}}$$ (3)

$$\begin{array}{cc} & D=d1+d2 \end{array}$$ (4)

**Fig 7:**
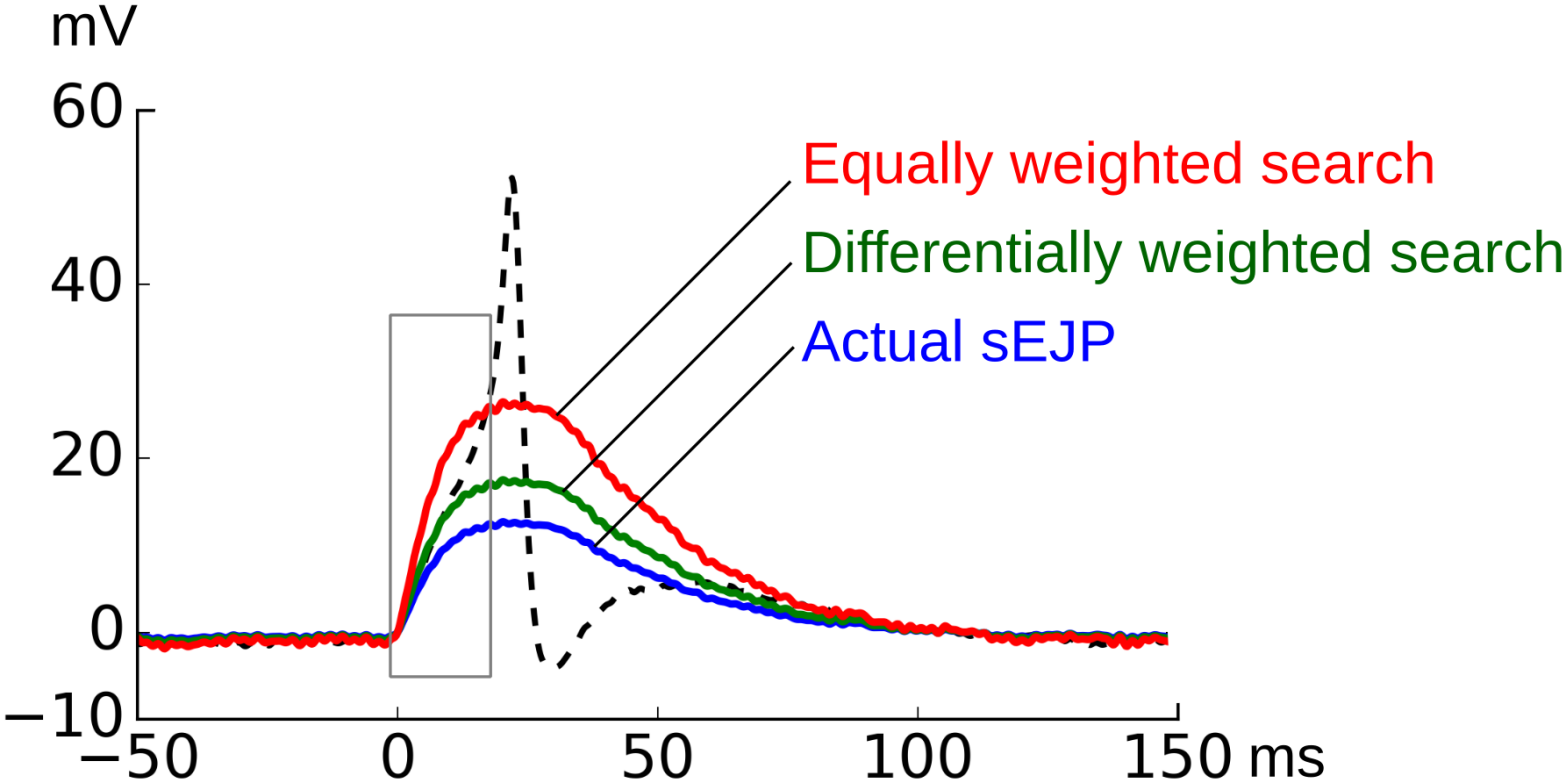
Amplification of the observed sEJP (solid lower trace, blue) to estimate the sEJP underlying AP. The amplification is determined by minimizing the distance measure. The amplified curves for equally weighted distance measure (solid upper trace, red) and the differentially weighted distance measure (solid middle trace, green) is also shown. The dotted line is the AP under consideration. The foot where the distance measurement was taken is shown in a box.

As can be noted, *d*2 as evaluated by the second method had a reduced weight over *d*1 and hence was less influential in deciding the multiplier values. This method gave a more robust sEJP multiplier estimate which produced the best approximation of the underlying sEJP for an AP, as shown in Fig 7.

#### 2.4.3 The estimation of nAP and sEJP prototype

Each AP belonging to the G0 group was compared with all the sEJPs present in the same cell of the AP. The sEJP which gave the minimum distance *D* was considered the best match sEJP for that particular AP. An estimate of the nAP was obtained from each AP by subtracting the respective best match sEJP after aligning their onsets. The process was repeated for several APs in an individual intracellular recording, and the best AP-sEJP pairs in that recording were shortlisted based on the normalized distance measure *D*_norm_ = *D*/*L* where *L* was the length of the AP foot. The mean value of the sEJP and nAP vectors of the shortlisted pairs were treated as the prototype sEJP and nAP shapes for that recording. Similarly, the prototype sEJP and nAP shapes were derived for all recordings available. Finally, the mean of the prototype shapes across the recordings were evaluated to obtain the universal sEJP and nAP prototype. The universal sEJP and nAP prototypes thus obtained were then normalized such that the baseline was at zero and the peak amplitude was unity.

### 2.5 Isolation of sAHP and vsAHP

The sAHP and vsAHP were isolated from G1 APs by comparing the APs which belong to G1 with those present in G0. The latter expresses sAHP or vsAHP along with the sEJP and nAP, while the former lacks the slow AHP components. As both the G0 and G1 APs contain the sEJP and nAP components, we posit that it may be possible to isolate the slow AHP component by subtracting the G0 AP from a matching G1 AP. For the sake of simplicity in isolating the components, it was assumed that sAHP and vsAHP events are mutually exclusive. With this assumption, every G1 AP have only three components—sEJP, nAP, and either sAHP or vsAHP. The G0 APs contain sEJP and nAP alone. Thus by subtracting the matching G0 AP from G1 AP, an estimate of the sAHP (and likewise vsAHP) was obtained.

A distance measure was designed to obtain the match between a G0 AP and a G1 AP. The matching operation was carried out across all available cell recordings. To avoid the effect of amplitude variations across the cells, the AP signals were normalized and peak-aligned before the comparison. The distance measure is described below in details.

#### 2.5.1 Distance measure between two APs

**Fig 8:**
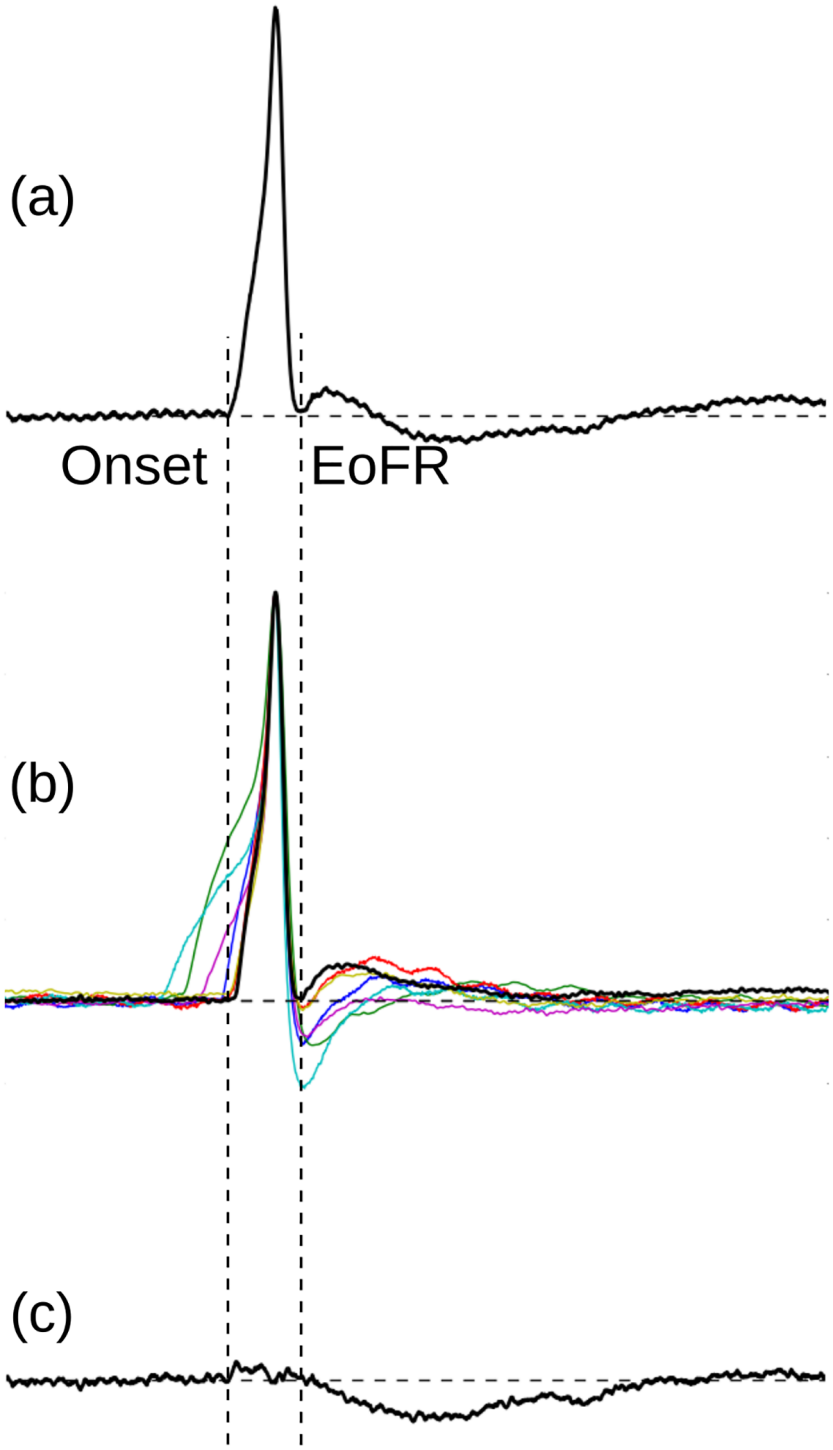
The extraction of slow AHP signal from a typical G1 AP. (a) The G1 AP from which the sAHP is to be extracted. The onset and end of first repolarization (EoFR) of the AP are marked. (b) The set of G0 APs which are peak aligned with G1 AP in (a) and matched against the Onset-EoFR window of G1 AP. The best matching AP is shown in a thicker trace. (c) The estimate of the sAHP component contained in the G1 AP obtained by subtracting the best matching G0 AP from the G1 AP.

The fixed signal here is the normalized G1 AP, and the variable signals were the G0 APs. The time span between the onset and the EoFR (the instance from which the sAP tail begins—the instance indicated by 2 in Fig 4) of the G1 AP is taken as the fixed window (*T* in Eq 5) in which the distance is measured (See Fig 8). Each of the normalized G0 APs were peak aligned with the G1 AP and the Euclidean distance (*D*) was measured between the two signals in the fixed window as given in Eq 5 below.

$$\begin{array}{c} D={\left|\right|V_{G1}\left(t\right)-V_{G0}\left(t\right)\left|\right|}_{t\in T} \end{array}$$ (5)

The G0 AP which yield the minimum value for *D* was taken as the closest match to the G1 AP under consideration.

#### 2.5.2 The estimation of sAHP and vsAHP prototype

Once the closest match for a G1 AP was obtained from the set of G0 APs, the matching G0 AP was subtracted from the corresponding G1 AP and the residual signal was examined. If the energy content of the residual signal in the matching window was comparable to the energy content of the noise, the pair of APs were declared to have a good match and the residual signal was considered as sAHP or vsAHP. This process was repeated for every G1 AP. The resulting residual signals were categorized into sAHP and vsAHP using an energy threshold. The signals below a threshold energy *E*_th_ was considered as sAHPs and the rest were declared to be vsAHPs. The threshold energy was defined as

$$\begin{array}{c} E_{\text{th}}=0.5\left(E_{\text{max}}+E_{\text{min}}\right) \end{array}$$ (6)

Where *E*_min_ and *E*_max_ were the minimum and maximum energy content in the residual signal pool respectively. Once the sAHP and vsAHP signals were extracted, the mean values of the shapes were obtained to derive a prototype shape of the sAHP and vsAHP. The resulting prototypes were normalized so that the baseline was zero and amplitude unity.

### 2.6 AP synthesis using extracted components

**Fig 9:**
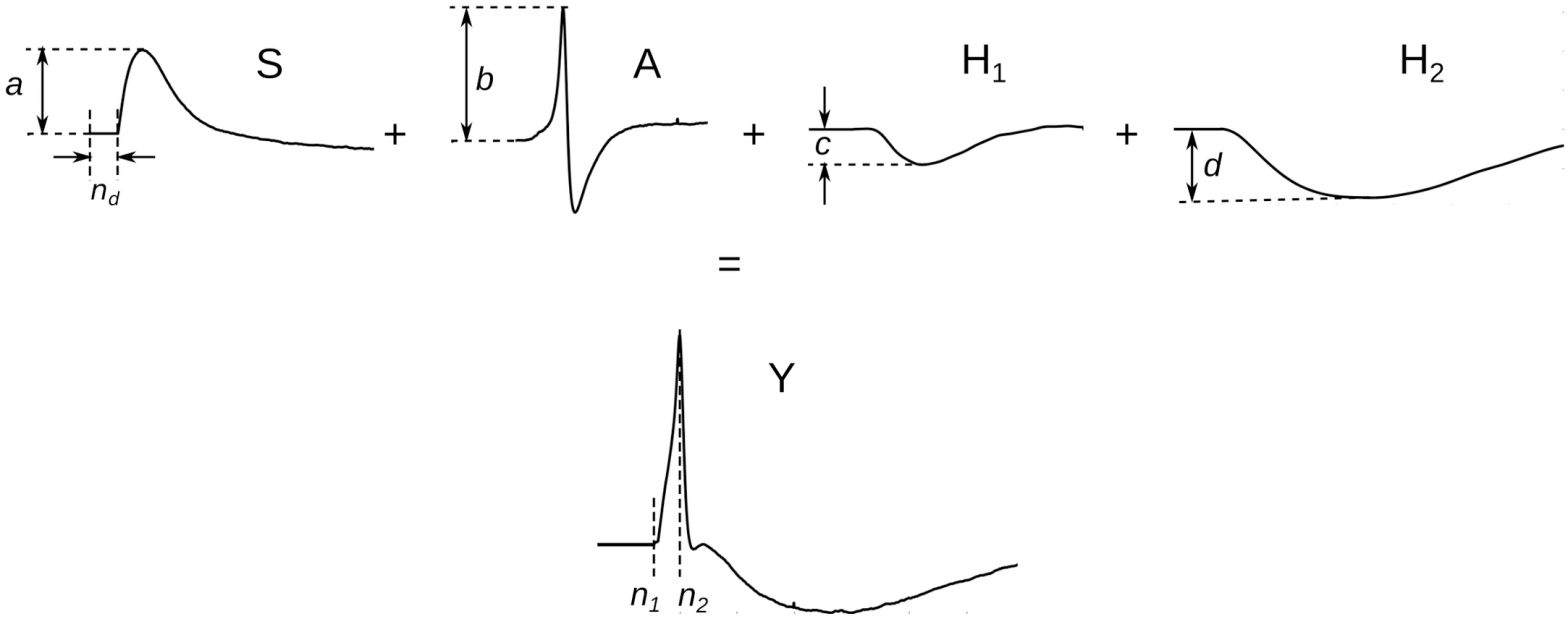
Schematic figure showing the synthesis of the AP using linear combination of the underlying components. The signals named *S*, *A*, *H*_1_, and *H*_2_, represent the sEJP, nAP, sAHP, and vsAHP components respectively. *n*_*d*_ is the time delay provided to the sEJP component relative to the other components. *a*, *b*, *c*, and *d* are the amplification factors assigned to each of the components. *Y* represents the output AP shape that is synthesized. *n*_1_ and *n*_2_ represent the time instants at which the onset and peak of the output AP occurs. In all practical cases, the condition *n*_*d*_ ∈ [*n*_1_, *n*_2_] holds true.

We hypothesized that a linear combination of the suitably processed/adjusted component templates should replicate various AP shapes observed. These adjustments of the templates include (i) amplification (ii) sEJP time-shift. The nAP, sAHP, and vsAHP components were aligned such that the starting point of sAHP and vsAHP components were in line with the nAP peak. A schematic figure showing the synthesis of a typical AP shape using the linear combination of the component template is given in Fig 9.

Thus, given an AP shape **Y**, it should be possible under our hypothesis to retrieve the sEJP time delay *n*_*d*_ and the amplification factors *a*, *b*, *c*, and *d* for each of the components such that the relation given in Eq 7 is satisfied.

$$\begin{array}{c} a.S\left(n-n_{d}\right)+b.A\left(n\right)+c.H_{1}\left(n\right)+d.H_{2}\left(n\right)\to Y\left(n\right) \end{array}$$ (7)

The vectors *S*, *A*, *H*_1_, and *H*_2_, represents the sEJP, nAP, sAHP, vsAHP components respectively. In order to estimate the unknown parameters, a vector based procedure was followed. The Eq 7 could be represented in the matrix form as

$$\begin{array}{lll} {\left[\begin{array}{cccc} S(1-n_{d}) & A(1) & H_{1}(1) & H_{2}(1)\\ S(2-n_{d}) & A(2) & H_{1}(2) & H_{2}(2)\\ .. & .. & .. & ..\\ S(N-n_{d}) & A(N) & H_{1}(N) & H_{2}(N) \end{array}\right]}_{N\times 4} & \times {\left[\begin{array}{c} a\\ b\\ c\\ d \end{array}\right]}_{4\times 1} & ={\left[\begin{array}{c} Y(1)\\ Y(2)\\ ..\\ Y(N) \end{array}\right]}_{N\times 1} \end{array}$$ (8)

or in short,

$$C_{n_{d}}X=Y$$ (9)

where $C_{n_{d}}$ is the component matrix in which each column represents each of the four AP component templates where **S** is given a time delay *n*_*d*_. **X** is the parameter matrix which contains the amplification factors corresponding to each of the component templates. **Y** represents the experimentally observed Type A AP shape which is to be approximated using the templates. For a given time delay *n*_*d*_, the amplification factor which best approximates the vector **Y** was obtained from

$$X_{n_{d}}={\left(C_{n_{d}}^{T}C_{n_{d}}\right)}^{-1}C_{n_{d}}^{T}Y$$ (10)

and the composite signal ${\hat{Y}}_{n_{d}}$ constructed using the estimated parameters corresponding to the sEJP delay *n*_*d*_, is given by

$${\hat{Y}}_{n_{d}}=C_{n_{d}}X_{n_{d}}$$ (11)

An iterative search operation was carried out to obtain the optimum value for *n*_*d*_. As indicated in Fig 9 the optimum value for the sEJP time delay *n*_*d*_ falls in the interval [*n*_1_, *n*_2_] where *n*_1_ and *n*_2_ are the time instants at which the onset and peak of the reference AP signal **Y** occurs. Hence the value of the delay *n*_*d*_ was varied across the interval [*n*_1_, *n*_2_] and for each *n*_*d*_, the corresponding composite vector ${\hat{Y}}_{n_{d}}$ was obtained. The value of *n*_*d*_ at which the Euclidean distance between **Y** and ${\hat{Y}}_{n_{d}}$ get minimized was considered as the optimum *n*_*d*_ and the corresponding parameter vector $X_{n_{d}}$ contained the optimum amplification factors.

### 2.7 Evaluation

The evaluation of the efficacy of the extracted components could be obtained by quantifying the ability of the components in replicating the various types of AP shapes observed in the recordings. The root mean square error (RMSE) was used as a measure of similarity between the recorded and the synthesized AP signals [22]. The RMSE between two signals $\bar{A}$ and $\bar{B}$, $D(\bar{A},\bar{B})$ was defined as

$$\begin{array}{c} D^{2}\left(\bar{A},\bar{B}\right)=\frac{1}{n}\sum_{k=1}^{n}{\left(a_{k-r}-b_{k}\right)}^{2} \end{array}$$ (12)

Where *D* = RMSE, *a*_*k*_ and *b*_*k*_ are the *k*^*th*^ samples of $\bar{A}$ and $\bar{B}$ respectively, *r* = the shift provided, in number of samples, to align the peaks of $\bar{A}$ and $\bar{B}$, and *n* = number of samples present in the active window of the signal $\bar{A}$. Active window of an AP is defined as the region of the signal between the onset and the end of signal (EoS—see Fig 4). If the RMSE between the recorded signal and its replication using the components was below a threshold value *T*, the approximation was declared a good fit. The value of *T* was estimated using the available AP signals as described below.

#### 2.7.1 Estimation of the RMSE threshold

**Fig 10:**
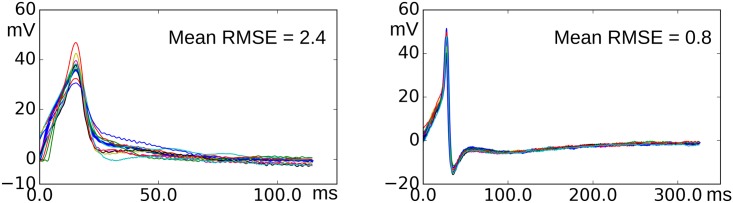
Overlapped plots of matching APs for two APs belonging to different classes. It can be observed that the variations in the matching APs given in the left panel is higher as compared to those in right. This difference is expressed in the root mean square error (RMSE) values given in the legend.

All APs belonging to the shortlisted cells for the training purpose were collected as a pool. For every such AP, the RMSE values were evaluated with respect to all other APs belonging to the pool. The values observed were arranged in the ascending order. The APs which produce the first 10 values in the list (addressed as the ‘matching APs’ for the current AP) would belong to the same AP class. It was assumed that the difference between two APs belonging to the same class was caused solely by the biological noise. The extent of noise observed varies significantly for APs belonging to different classes, as shown in the Fig 10. Hence a separate threshold value was defined for different AP classes, instead of a single universal RMSE threshold. For *i*^*th*^ AP in the pool, a specific RMSE threshold *τ*_*i*_ was defined as the maximum of the RMSE values measured between the *i*^*th*^ AP and its matching APs. Also a universal RMSE value *T* was defined as the mean of all specific RMSE values in the pool. The universal RMSE value could be used to check the goodness of fit for an AP signal which does not belong to the training data used in this study in which case the specific RMSE threshold is not available.

## 3 Results

### 3.1 Selection of cells

We obtained 23 intracellular recording sessions from 20 animals. During a session, once the electrode penetrates the smooth muscle cell, the recorded potential drops from zero to the resting membrane potential (RMP). It takes about 5 to 10 minutes for the cells to stabilize after the impalement. The duration of recording mentioned in the manuscript represent the duration of stable recording only, excluding the initial period. In a single session, it was possible to have recordings from multiple cells, each of which are termed ‘cell recordings’. Thus we had 73 cell recordings available for analysis. These recordings were divided into two sets—training set and the test set. The training set was used to obtain the model components. In order to train the model for obtaining the best estimates of the components, it was required to have as many number of sAPs and sEJPs as possible, so they can be paired up in order to find the best matches. However, such long electrophysiological recordings which contain large numbers of sEJPs and sAPs are difficult to obtain and hence are highly limited in number. Hence a set of criteria were adapted such that we could maximize the number of signals in the recordings and at the same time maximize the number of cells shortlisted for training. Thus the following three criteria were set to include a cell recording to the training set: 1) The recording should be at least 5 minutes in duration 2) it should contain at least five Type A APs and 3) it should contain at least 5 sEJPs with amplitude > 5 mV. The training set include 26 cell recordings which satisfied the aforementioned conditions.

Once the components were identified and the model was fixed, there were no restrictions imposed on the number of APs utilized in the testing phase. The testing phase requires only individual sAPs, and no pairing-up is needed. For example, even if there is just one sAP present in the entire recording, its components can be estimated. Hence the rest of the cells were allotted to the test set. The summary of the activity statistics present in the training and test set are given in Table 1. It can be observed that there is an uneven distribution of signals between the training and test sets. This is expected because of the criteria we have used to allocate the cells to the training set. Because of the set criteria, all the long duration recordings which contain large numbers of Type A sAPs and sEJPs were added to the training set. The rest of the recordings were generally shorter, with lesser number of Type A sAPS and sEJPs, but a larger share of Type B sAPs, causing the uneven distribution.

**Table 1:** The pooled summary of different types of signals available in the cell recordings used in the study.

Pool	#Cells	#AP (Type A)	#AP (Type B)	#sEJP	Av. Dur.
Training Set	26	934	79	2422	33.97 min
Test Set	47	266	166	466	12.91 min

### 3.2 General observations

The RMP values of the cells were determined as an average of the membrane potentials preceding the onset of the individual activities. The values thus obtained ranged between −28 and −64 mV (−43 ± 7 mV). There was a small difference in the resting membrane potential between the training and test set (−41 ± 6 versus −45 ± 7 mV respectively), and this might explain the small differences in the sAP frequency distribution between the two sets.

The standard parameters of sAPs and sEJPs in training and test set are now compared. The parameters used for sAPs were amplitude, half-width, and AP duration whereas those used for comparison of STDs were amplitude, rise time, and fall time. Average values obtained for all signals belonging to training and test set are given in Table 2. From the table, it can be observed that all the parameters, except sAP amplitude, have similar values for training and test sets (*p* > 0.05). The sAP amplitude for training set is found to be higher than that for test set (*p* < 0.01). This may be attributed to the difference in the RMP values. As mentioned earlier. the test set recordings have a slightly elevated resting potential compared to the training set. The sAP signals originating from a depolarized membrane will have a lower amplitude because (a) a greater number of L-type Ca channels will be in the inactivated state, causing a reduced inward current during sAP generation, and (b) more K channels will be activated, which increases the outward current thus reducing the peak of the sAP.

**Table 2:** The comparison of standard features of signals observed from the training and test sets. The sAP (Type A only) amplitudes exhibit smaller amplitudes in the test set compared to the training set (*p* < 0.01—marked ‘**’ in the table), the reason for which is explained in the text. Rest of the parameters have comparable values for training and test sets (*p* > 0.05). The parameter values are given as mean ± SD. Number of signals used in Training set: 2487 sEJPs and 1013 sAPs; in Test set: 477 sEJPs and 453 sAPs.

Signal Type	Parameter	Training Set	Test Set
sEJP	Amplitude (mV)	9.23 ± 3.56	9.08 ± 3.11
	Rise Time (ms)	22.29 ± 13.86	22.57 ± 15.13
	Fall Time (ms)	9.23 ± 3.56	9.08 ± 3.11
sAP	Amplitude (mV) **	52.49 ± 7.60	49.33 ± 9.27
	Half Width (ms)	9.62 ± 4.03	9.83 ± 4.99
	AP Duration (ms)	164.47 ± 81.58	165.70 ± 97.04

### 3.3 Isolation of sEJP and nAP components

The baseline segments in all AP signals in the training set were examined and the maximum standard deviation of the noise present at the baseline ($max(\sigma_{n_{i}})$) was evaluated to be 0.91 mV. Thus the threshold value for onset detection of the signals were set as 1 mV above the RMP of the corresponding signal. This onset threshold was kept fixed for all the studies present in this work.

**Fig 11:**
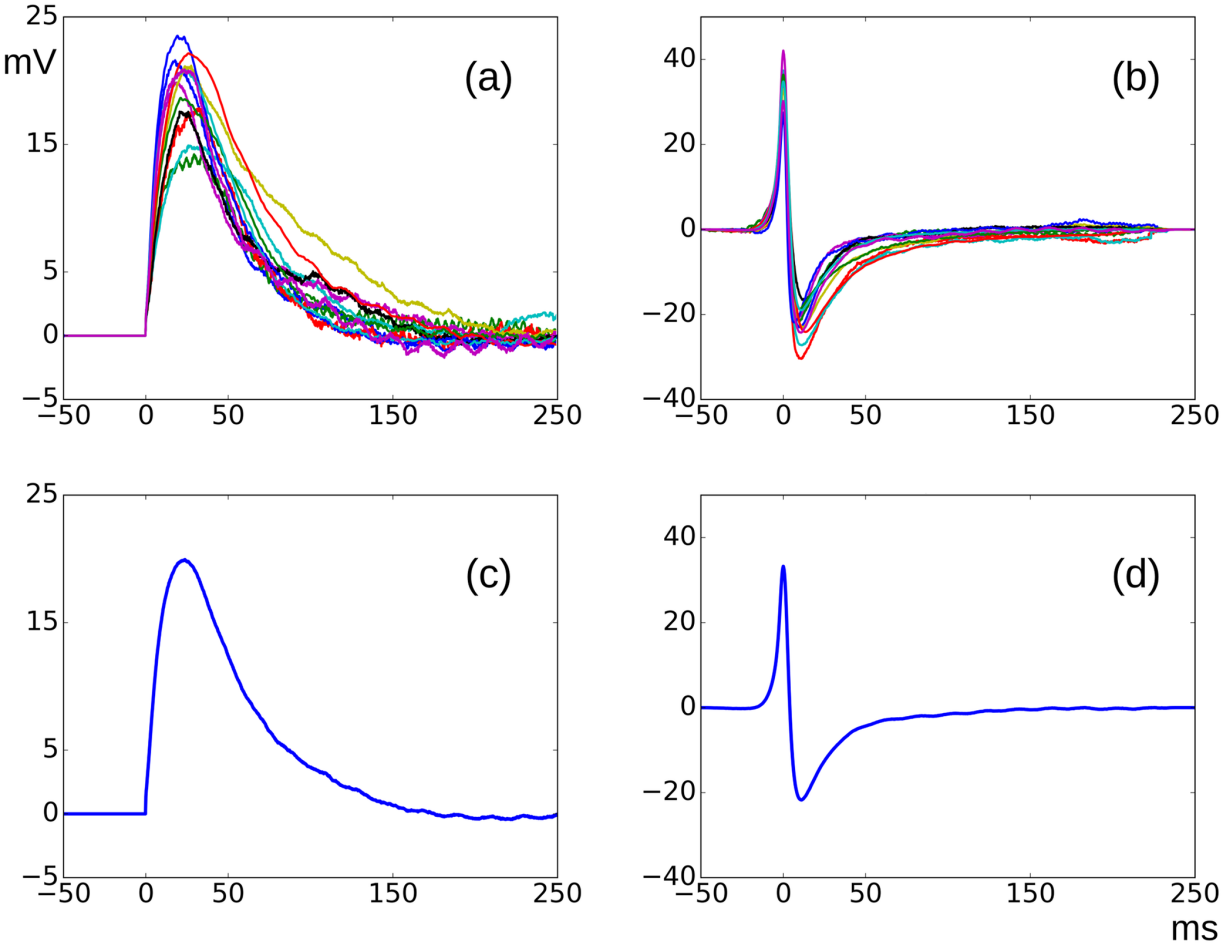
Top: Overlaid plots of cell wise sEJP (a) and nAP (b) templates obtained from the shortlisted sEJP-AP pairs belonging to individual cells. Only the cells with more than 4 shortlisted sEJP-AP pairs are shown. Bottom: The sEJP (c) and nAP (d) templates derived from all the sEJP-AP pairs shortlisted from the training set.

For each of the G0 AP signals belonging to the training set, the best matching sEJP from the corresponding cell was obtained as described in the Section 2.4. The matching AP-sEJP pairs thus obtained were then visually inspected and the erroneous matches were rejected. After this operation, there were 12 cells which contained at least 4 acceptable AP-sEJP pairs. The onset-aligned best-matching sEJP was subtracted from the corresponding AP to obtain the native AP. The mean of all the the best matching sEJPs and the native APs thus obtained from the shortlisted sEJP-AP pairs for individual cells are given in the Fig 11(a) and 11(b) respectively. The mean of the pooled best matching sEJPs and the resultant nAPs across all the cells were taken as the universal sEJP and nAP templates, which are shown in (c) and (d) of Fig 11. These templates were amplitude normalized and were saved in text files and later were used for the synthesis of the AP signals.

### 3.4 Isolation of sAHP and vsAHP components

**Fig 12:**
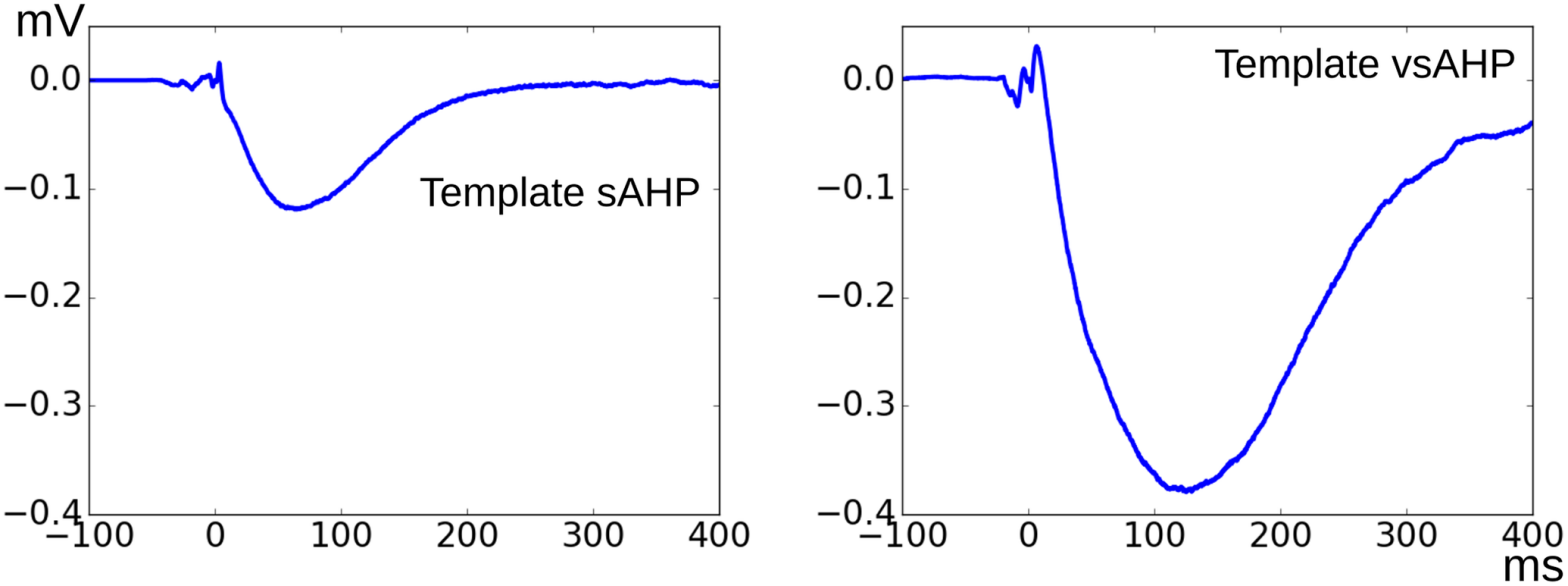
The sAHP (left) and vsAHP (right) templates derived from all the shortlisted AP-AP pairs observed from the training set.

Type A APs in the training set (total 934) were pooled and were separated into two groups—Group 0 (G0) and Group 1 (G1) APs—which contained 544 and 390 signals respectively. For every AP in Group 1, a search operation was carried out as described in Section 2.5 to get the best matching AP from the G0 pool. The obtained AP-AP pairs were then visually examined and the erroneous matches were discarded. Thus there were 134 shortlisted AP-AP pairs with good match in which 104 pairs represent the G1 APs showing sAHP tail and the rest were those exhibiting the vsAHP. From every AP-AP pair thus obtained, the peak-aligned G0 AP is subtracted from the G1 AP to obtain an estimate of the sAHP/vsAHP component. The ensemble average of all the sAHP components thus obtained was finalized as the sAHP template. Similarly the vsAHP template also was obtained. The estimated sAHP and vsAHP templates are shown in the Fig 12. These templates were normalized so the maximum negative amplitude is unity and were saved in text files to be used in the AP synthesis.

### 3.5 AP synthesis using component templates

**Fig 13:**
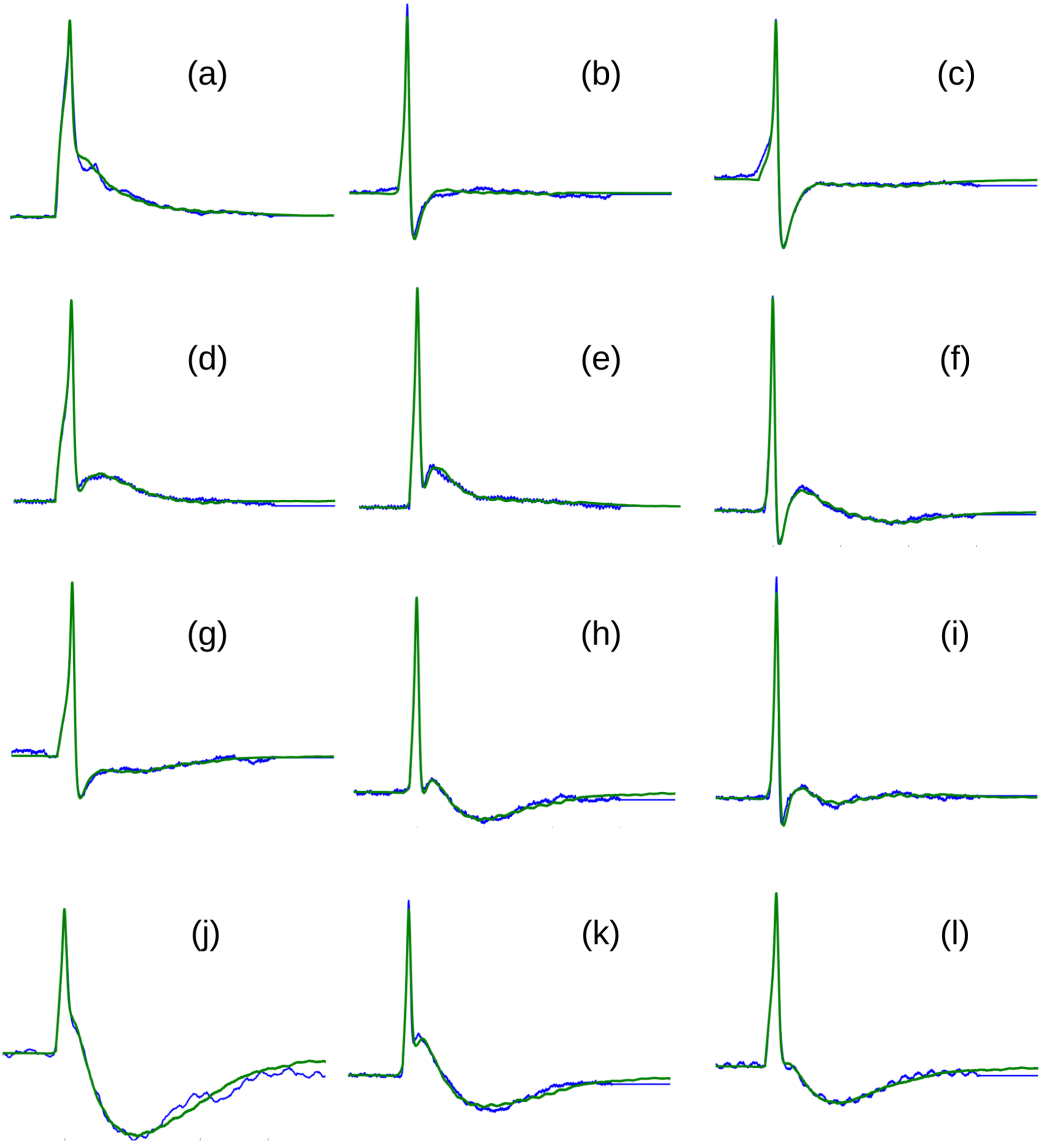
The examples of good replications of the experimentally obtained intracellular sAPs obtained using the proposed 4-component model. The traces in blue are the recorded signals, and the green traces are the synthesized signals. Only the sAPS belonging to the Test set are shown.

For each of the sAP signals, in both training and test sets, Eqs 10 and 11 were used to obtain the ideal time shift and the amplification factors for generating the closest matching synthetic AP. The peak aligned plots of the recorded AP signals and the corresponding synthetic AP signals produced by the component templates were visually examined and found that most of the AP signals were replicated satisfactorily. Some examples of the match between the different classes of AP signals belonging to the test set and the corresponding synthetic signal are shown in Fig 13 It can be noted that almost all variations observed in the Type A sAP signals were captured by the proposed 4-component model.

### 3.6 Evaluation of the goodness of fit

All APs belonging to the training set (total 1013 including Type A and Type B) were pooled together, and for each of those APs, 10 closest matching APs were shortlisted from the pool. The matching APs were visually examined to ensure that all the 10 matching APs belong to the same shape category. Two exceptions were found which were removed. For every AP that remained, the RMSE values between that and each of the matching APs were obtained. The maximum RMSE value among the 10 matching APs was chosen as the specific RMSE value for that corresponding AP. The specific RMSE value in the training set varied between 0.93 and 9.15 with a mean value of 2.34 ± 0.81. The mean value was set as the universal RMSE value *T* for further analysis.

Every AP signal belonging to the test set was replicated using the component templates. The RMSE values between the recorded APs and the corresponding synthesized APs was measured. If the RMSE value fell below *T*, the synthesized AP was deemed a good fit. The overall replication efficiency was obtained as the percentage of the AP signals which were replicated by the components with a good fit. For the test set, the replication efficiency of 63% was obtained (Table 3).

**Table 3:** The replication efficiencies obtained for different data sets used in the study.

Data Set	Total	# Good Fit	R. efficiency
Training Set	934	654	70%
Test Set	266	168	63%

## 4 Discussion

The initial electrophysiological studies on the detrusor SMCs were conducted on rabbits [8, 23] and guinea-pigs [9, 13, 14, 24]. It was observed that guinea-pig bladder exhibit rhythmic spontaneous AP signals. The action potentials recorded exhibit neither any observable convexity at the foot nor any ADP. Also those recordings seldom contained sEJPs—which is an indication that those spontaneous APs have a myogenic origin [13, 14]. The activity level of the rabbit bladder varied from cell to cell. Some cells produced rhythmic spontaneous APs like in guinea-pig bladder and some were silent [8, 23]. In mouse urinary bladder DSM, it was observed that the spontaneous activities occur randomly in time and are predominantly neurogenic in origin [10–12]. Thus the properties of the detrusor tissue vary between species. Across species, pharmacological studies indicated that the falling phase and AHP of the APs are mediated by the big and small conductance (BK and SK) Ca^2+^-activated K channels respectively [9, 12, 13]. The role of K^+^ channels in Mouse DSM is extensively studied [10–12, 15, 25]. There is some evidence that the SK and voltage-gated K^+^ channels (K_*V*_ channels) contribute to the slow AHP signals [12, 15, 25]. In a recent study by [21], it was shown that the SK channels contribute to the duration of the sAHP signals in guinea-pig DSMC. However, the lack of variability of the K_*V*_ channel activity fail to explain the stochastic appearance of the sAHP feature in sAPs, and the regulatory aspects of the SK channels still remain unknown [15].

Our method presents a novel approach which could serve as a support to explore the mechanisms behind the shape variability of the sAPs seen in the mouse DSMCs. Here we identified four basic components which are necessary and sufficient for explaining all the sAP shapes seen in the experimental recordings. The assumptions used to build the model, physiological significance of two of the hyperpolarizing components, and the implications and applications of the proposed model are discussed in the following subsections.

### 4.1 Choice of the mouse model

We have chosen to explore the mouse bladder because of the more detailed molecular knowledge of its channel populations, and the presence of purinergic transmission, noting that aging human bladder displays a high degree of purinergic activity [10, 26–29]. Some studies have also indicated that purinergic neurotransmission also plays a role in normal human bladder voiding [30]. Another aspect in which the mouse bladder is similar to the human bladder is the existence of intramural ganglia [31, 32]. It has been found that the intramural ganglia are absent in other species such as adult rat bladder [33, 34]. Apart from the abovementioned physiological similarities, the ease of doing gene-knockout studies in mice [11, 12, 25] makes it amenable to detailed studies on putative mechanisms operating in the bladder tissue.

However, while up-regulation of purinergic neurotransmission—known as atropine-resistance—can be seen in pathological human bladder [26, 35], increased AP firing triggered by purinergic sEJPs in pathological human bladder has not yet been demonstrated. A limitation of this model is that the electrical properties of mouse DSM are not very similar to those in other species including human in terms of the origin of AP generation.

### 4.2 Assumptions used and technique development

It is observed neuronal synapses that neurotransmitter action generating a spike through an EPSP will continue to exist after the spike and will be seen as a hump or an after depolarization on the falling phase of the spike [36]. A similar observation is made at neuro-muscular junctions where action potentials recorded near neurotransmitter release points exhibit a significant convexity at the AP foot and also display a hump, observable during the falling phase of the AP [20]. We assumed that the same phenomenon could underlie the convex foot and ADP observed frequently in the sAP signals of the mouse DSMC. sEJPs observed in the smooth muscle cells are the equivalent of mEPSPs in neuron and mEPPs in skeletal muscles. These can be observed occurring in the absence of nerve stimulation. While mEPSPs and mEPPs are low-amplitude events, sEJPs can possess high amplitudes capable of crossing the threshold and thus producing action potentials in the smooth muscle cells. These high amplitude sEJP signals usually are masked by the AP but can be observed if the action potentials are abolished by blocking the voltage gated Ca^2+^ channels using nifedipine [10]. However it is not possible, even by any pharmacological means, to observe the native AP shape which is produced by the voltage and Ca^2+^ channels i.e., cell’s internal active mechanisms to generate the AP. The reason behind this is the necessity of a threshold membrane voltage which is required to trigger the regenerative mechanisms driven by the voltage gated channels. Since their threshold voltage is supplied by sEJPs that attain the critical amplitude, the AP always rides upon the sEJPs, as at skeletal neuro-muscular junctions where AP rides upon EPP. The closest approximation to the native AP is the traveling AP recorded at a considerable distance away from the source DSMC. However in a heavily innervated, loosely coupled mouse DSMC, such traveling APs are not commonly observed. Hence the only way by which an estimate of the native AP can be obtained, is by subtracting the underlying ligand-gated passive sEJP signal from the composite sAP, assuming that there is a linear superposition of the two signals. Owing to the above mentioned reasons, the nAP signals are not observed on their own. They vary in amplitude, depending on the underlying sEJP amplitude. The active channels adjust their currents such that the amplitude of the sAP vary only in a relatively narrow window. However, it was observed that the shape of the estimated nAP remains the same after amplitude normalization (Fig 11). This observation indicates that it is reasonable to make an assumption that there exists a linear superposition between the passive sEJP and the active nAP signals. This method could be extended in a similar manner to obtain the sAHP and the vsAHP signals, and any further components, if observed.

A differential-weight distance evaluation is used for quantifying the match between the sAP and the sEJP signals. The first derivative of the rising phase of the Type A APs (neurogenic sAPs) exhibit double peaks [10]. The first peak indicates the onset of the neurotransmitter action and the second peak indicates the operation of the regenerative ionic mechanisms that generate the sAPs. The transition from passive to active behavior of the membrane occurs gradually during the time interval between the first and second peak of the first derivative. The instant at which the first derivative reaches the lowest value between the two peaks is considered as the end of the foot (EoF, Figs 4 and 7) [18]. There is no clear demarcation between the passive and active region of the AP and the active behavior becomes more prominent towards the end of the foot. This property of the foot is addressed in the distance measure used (Eq 4) which attributes a lesser weight to the elements towards the end of the foot.

Robust statistical tests for checking the goodness of fit between the experimentally recorded and synthetic signals are not available [37]. However, quantification of the fit of the synthetic signals to the experimental signal could be performed using some normalized distance measurement. In the present study, a normalized Euclidean distance measure termed RMSE was used for quantifying the fit. This method is commonly used in the field of proteomics to decide the goodness of match between protein structures [22]. If the RMSE measure is below a certain threshold, the signals can be deemed a good match. The choice of the threshold RMSE value is critical, and entails an element of subjectivity. To reduce the subjectivity, we used a large size of training data to decide the threshold value. From the shortlisted pool of 934 neurogenic AP signals present in the training data, a threshold value of 2.34 was chosen, as described in the Section 2.7.1. The threshold thus set ensured that the match between each of the sAP signals in the training set and the majority of the AP signals present in the corresponding ‘matching AP’ set were identified as a good match. Thus if the synthetic AP signal is declared as a good fit to the experimental signal, the difference between them falls within the biological variability observed among the sAP signals present in the training data set. The efficiency of the RMSE measure is maximized by restricting the window of measurement as the region between onset and the EoS of the experimental curve. The fairly good success of the model (63%) in replicating the experimental signals supports the assumptions made in the model development, such as the identification of the 4 components and their linear superposition, at least as a first line of approximation.

### 4.3 Physiological significance of the sAHP and vsAHP components

A combination of slow and fast potentials that sum to give complex electrical signals is observed at other synapses also. For example, a slow hyperpolarizing IPSP is observed in C cells of the bullfrog sympathetic ganglia, principal cells of the mud puppy parasympathetic ganglion cells and in the associated cardiac cells [36, 38–40]. The onset of these IPSPs occurs at a latency of 50-100 ms after the release of neurotransmitter from the presynaptic terminals, and in time course they last for around a second. These signals are caused by the activation of the muscarinic ACh receptors, and are mediated by K^+^ ions alone. Such IPSPs are not abolished by the administration of curare and hence are not associated with the nicotinic EPSPs and do not inhibit the APs generated by those EPSPs [39]. In comparison, the properties of these IPSPs resemble the slow hyperpolarization signals observed in the DSM cells.

Though several studies have been conducted on the sAHP component of the sAPs, little attention was paid to the very slow AHPs, probably because of their rare appearance. They occur only in 14 out of 73 cell recordings used in this study (combining both the training and test set). When they appear, their percentage contribution to the overall cell activities is very low, varying between 0.5% and a maximum of 2.7%, with a mean of 1.6%. As the time course of the vsAHP is comparable to that of the calcium waves seen in the mouse DSM syncytium, it could be hypothesized that the vsAHP feature is produced by the activation of one or more of the Ca^2+^-dependent ion channels, known to be present in the DSM cells.

**Fig 14:**
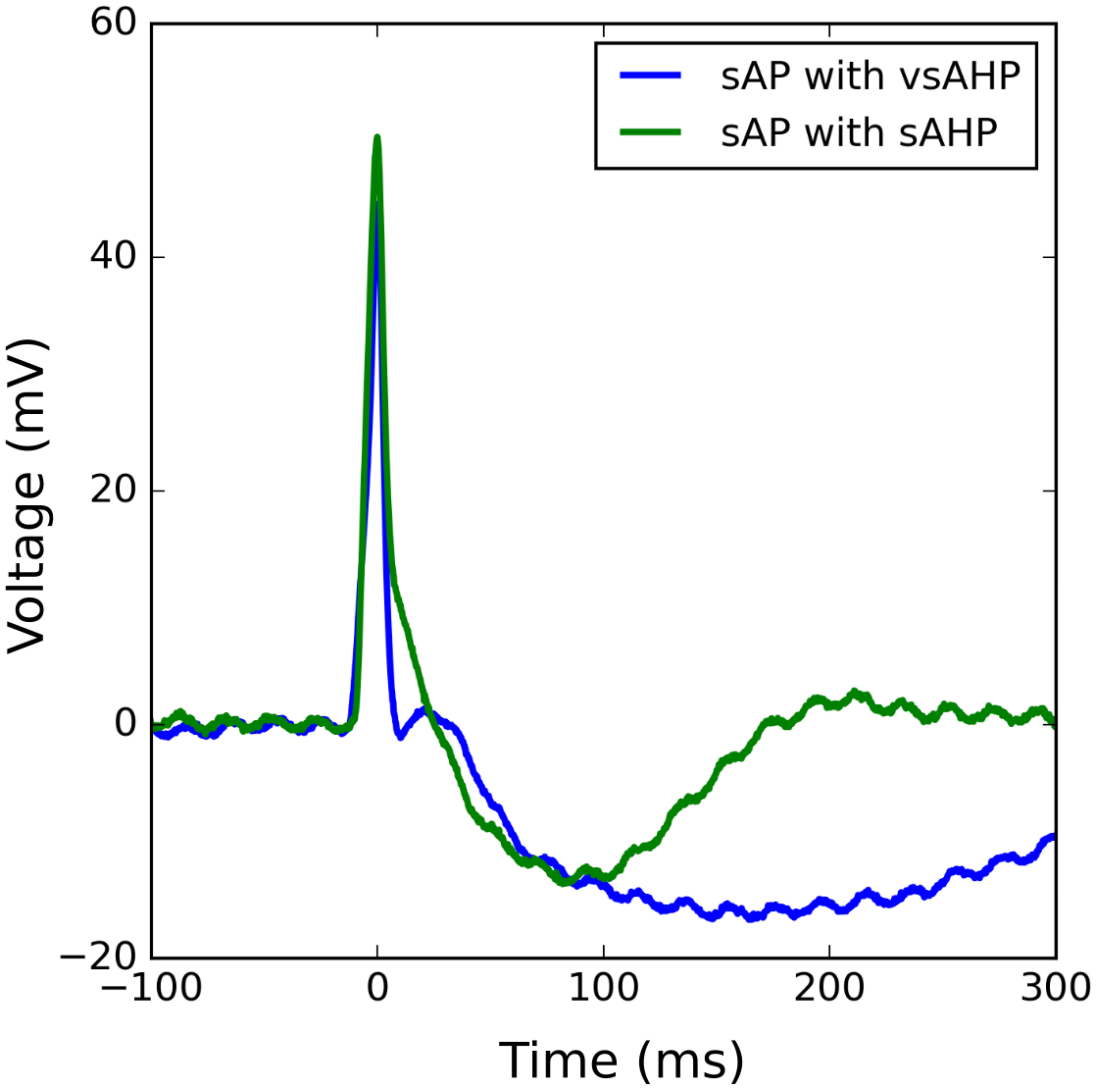
An example instance where the amplitudes of the sAHP of an sAP signal is comparable to that of the vsAHP component of another sAP. Both sAPs are recorded from the same cell. This indicates that the vsAHP component is not merely a prolonged version of the sAHP component.

A question might arise on the separability of the sAHP and vsAHP components, as to whether the vsAHP is merely an extended version of the sAHP component, and hence the two should be treated as a single component. This can be addressed as follows. Though it is generally observed that the vsAHPs are of much larger amplitude compared to that of sAHPs, there are cases where the amplitude of the sAHP is comparable to that of the vsAHP. One such example is shown in Fig 14. This indicates that the vsAHP is not merely an amplified/prolonged version of the sAHP, and hence there might exist distinct underlying mechanisms which cause the two types of AHPs. However, such an inference is not conclusive because the separation of sAHP and vsAHP by ionic conductance or pharmacological blockade has not been proven. Thus, sAHP and vsAHP could be the same phenomenon displaying quantitative but not qualitative difference.

Another major reason for keeping two separate components in representing the slow afterhyperpolarizations in the sAPs was to account for the variability in their time courses. If an attempt was made to combine the sAHP and vsAHP into a single component and make a three-component model of the detrusor sAP, it would not be possible to satisfactorily replicate the time course of the slow afterhyperpolarizations without arbitrarily stretching or compressing the third component in time.

### 4.4 Type A and Type B sAPs

As mentioned in the introduction, Type A sAPs are thought to be of neurogenic origin and Type B sAPs are thought to be of myogenic origin [10–12]. It is established that the source of Type A sAPs are the varicosities of the innervating neurons, whereas the origin of Type B sAPs is unknown.

**Fig 15:**
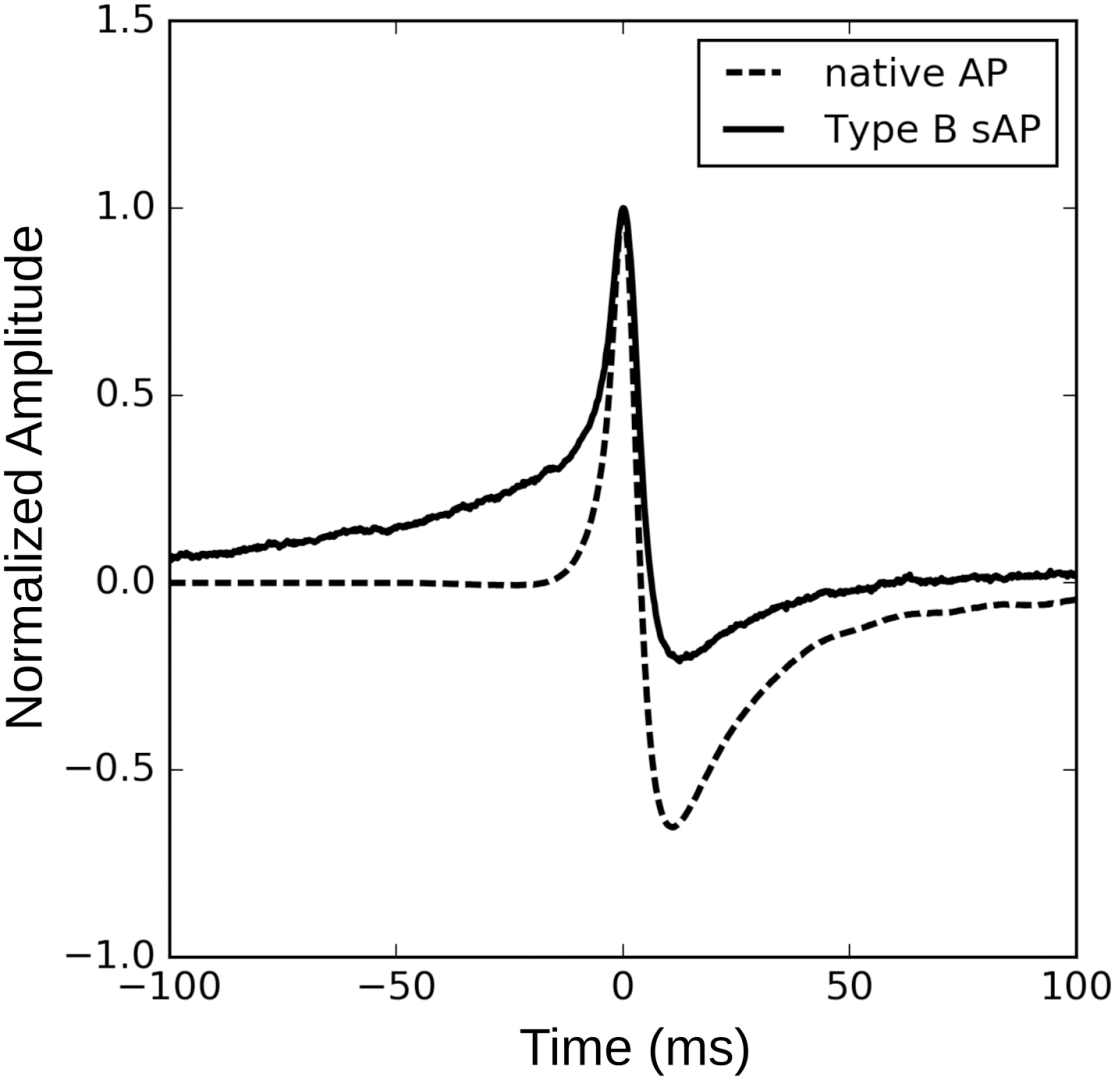
Overlaid plots of a typical Type B sAP (solid line) and the native AP template obtained from the Type A sAP decomposition (dashed line). The amplitudes are normalized and the peaks aligned at time = 0 ms. Note the slow ramp component which is a signature component of Type B sAP.

Though the Type B sAPs do not have an sEJP component, they cannot be considered as native APs (nAP). The difference between the nAP template obtained from our analysis of the Type A sAPs and a typical Type B sAP is evident from the superimposed plot shown in Fig 15. The amplitudes are normalized and the peaks aligned at time = 0 ms. The Type B sAP exhibits a ramp foot roughly 100 ms in duration before it reaches threshold and initiates the AP. This ramp foot is a signature of the pacemaking type AP and is absent in the native AP, which reaches the threshold well within 20 ms after the onset. Thus it may be assumed that the Type B sAPs consist of (at least) two components—1) a ramp component which raises the membrane potential gradually to threshold, and 2) the native AP which may be similar to that present in the Type A sAPs. Since the origin of the ramp of Type B sAPs is not delineated, we are not yet in a position to investigate the properties of the ramp and hence are unable at present to separate the ramp from the composite Type B sAP.

The aforesaid points constitute the reasons why the Type B sAP cannot be considered as the native AP. In fact, once more data are available, a separate analysis of Type B sAPs needs to be done on its own merits, which stands to shed light on the component signals that constitute these sAPs and the mechanisms of their generation.

Irrespective of the origin of these different types of sAPs, they are known to play a critical part in the generation of smooth muscle tone in the detrusor, arising from micromotions set off by the spontaneous APs [41]. Therefore, detrusor tone could be affected by changes in either Type A or Type B sAPs. Changes in Type A sAPs would then primarily affect the “neurogenic tone” and changes in Type B sAPs would primarily affect the “myogenic tone”. In either case, a better understanding of the origin and properties of the sAPs, for instance in terms of their constituent components, would lead to a better understanding of the physiological underpinnings of detrusor tone and the disorders arising from disrupted tone.

### 4.5 Implications of the model

From work presented here, it can be observed that the four components extracted from the experimental signals are necessary to explain all the different shapes seen in the neurogenic APs. From the reconstruction studies, it can be observed that these four components are not just necessary but also sufficient to satisfactorily replicate all the Type A APs observed in the mouse DSM cells. This implies that these four components signify four separate sub-signals which underly the spontaneous APs generated by the neurotransmitter release. This finding could be used as a cue for devising further experimental studies to explore, with better clarity, the mechanisms behind the electrical activities of mouse DSMCs. For example, our study show that not all the cells display sAHP and vsAHP components. This could suggest that there exist distinct population of cells that either display or do not display the slow AHPs and, this in turn may imply that there may exist distinct populations that subserve distinct functions. The longer the duration and amplitude of the AHP, the lower will be the frequency of APs producible by the cells. Hence the presence and absence of the vs/sAHP components might be the means to differentiate the SM cells that are teleologically purposed to fire at lower frequency or, in mechanical terms, to contract more slowly. Cells in DSM are known to be organized in bundles, which display structural heterogeneity. Moreover, bundles are present in varying orientations in the detrusor. It is possible that certain bundles may be endowed with functional characteristics as dictated by the presence or absence of vs/sAHPs components in the APs produced in their constituent cells. Our prediction is amenable to being tested experimentally, for instance simultaneous recordings and calcium imaging as already performed by Young, Meng, and Brain [10, 11].

### 4.6 Applications of the model

The technique used in this work to isolate the components of the DSMC could be generalized for similar applications to other biological signals. Other than mouse DSM, significant variations in the AP shapes generated by a single cell type in a tissue is not observed. However, in other peripheral composite signals such as the visual, auditory, or somatosensory evoked potentials, there is a need to detect and isolate the individual events [42–45] where the proposed technique may be useful.

Apart from the identification of the physiological processes behind various features of the AP signals observed, the 4 component model also provides a means to classify (and compress) the sAPs. Any neurogenic AP in the DSMC can now be represented by 5 parameter values, namely, the amplification factors for the 4 component templates (*a*, *b*, *c*, *d*) and the time delay (*n*_*d*_) of the sEJP component. Those parameters could be used for the classification of the AP signals, which would be helpful in understanding the properties of the DSMC and its surroundings. Other methods of classification, such as the K-Means [16], Hierarchical [19], or feature-based classifications [18], require heavy computation time and also are error-prone. The use of the parameters obtained from the model could improve the efficiency of the classification schemes.
